# Response of biotic communities to salinity changes in a Mediterranean hypersaline stream

**DOI:** 10.1186/1746-1448-2-12

**Published:** 2006-10-02

**Authors:** Josefa Velasco, Andrés Millán, Juan Hernández, Cayetano Gutiérrez, Pedro Abellán, David Sánchez, Mar Ruiz

**Affiliations:** 1 Department of Ecology and Hydrology, University of Murcia, 30100 Murcia, Spain

## Abstract

**Background:**

This study investigates the relationship between salinity and biotic communities (primary producers and macroinvertebrates) in Rambla Salada, a Mediterranean hypersaline stream in SE Spain. Since the 1980's, the mean salinity of the stream has fallen from about 100 g L^-1 ^to 35.5 g L^-1^, due to intensive irrigated agriculture in the watershed. Furthermore, large dilutions occur occasionally when the water irrigation channel suffers cracks.

**Results:**

Along the salinity gradient studied (3.5 – 76.4 g L^-1^) *Cladophora glomerata *and *Ruppia maritima *biomass decreased with increasing salinity, while the biomass of epipelic algae increased. Diptera and Coleoptera species dominated the community both in disturbed as in re-established conditions. Most macroinvertebrates species found in Rambla Salada stream are euryhaline species with a broad range of salinity tolerance. Eight of them were recorded in natural hypersaline conditions (~100 g L^-1^) prior to important change in land use of the watershed: *Ephydra flavipes, Stratyomis longicornis*, *Nebrioporus ceresyi*, *N. baeticus*, *Berosus hispanicus*, *Enochrus falcarius*, *Ochthebius cuprescens *and *Sigara selecta*. However, other species recorded in the past, such as *Ochthebius glaber*, *O. notabilis *and *Enochrus politus*, were restricted to a hypersaline source or absent from Rambla Salada. The dilution of salinity to 3.5 – 6.8 gL^-1 ^allowed the colonization of species with low salininty tolerance, such as *Melanopsis praemorsa*, *Anax *sp., Simulidae, Ceratopogonidae and Tanypodinae. The abundance of *Ephydra flavipes *and *Ochthebius corrugatus *showed a positive significant response to salinity, while *Anax *sp., *Simulidae*, *S. selecta*, *N. ceresyi*, *N. baeticus*, and *B. hispanicus *showed significant negative correlations. The number of total macroinvertebrate taxa, Diptera and Coleoptera species, number of families, Margalef's index and Shannon's diversity index decreased with increasing salinity. However, the rest of community parameters, such as the abundance of individuals, evenness and Simpson's index, showed no significant response to changes in salinity. Classification and ordination analysis revealed major differences in macroinvertebrate community structure between hypersaline conditions (76.4 g L^-1^) and the rest of the communities observed at the lower salinity levels, and revealed that below ~75 g L^-1^, dissimilarities in the communities were greater between the two habitats studied (runs and pools) than between salinity levels.

**Conclusion:**

Salinity was the first factor determining community composition and structure in Rambla Salada stream followed by the type of habitat.

## Background

Saline inland waters (salinity ≥ 3 g L^-1^) are geographically widespread, especially in arid and semi-arid regions of the world. Such habitats are globally threatened by human activities that induce changes in the natural hydrology and salinity levels, with a corresponding loss of biodiversity [1]. Hydrological alteration and associated changes in salinity in aquatic systems are frequently associated to changes in land use in the corresponding watershed [2,3]. Natural salt lakes have been the focus of most limnological studies of saline waters. Environmental threats to salt lakes have been well documented [1], but those to saline streams, especially hypersaline streams, have been poorly studied. This is a pity because hypersaline running waters are particularly interesting due to their halotolerant/halophilic biota and the rarity and the high number of endemic species that they sustain: for example, some species of aquatic Coleoptera [4-6], which often have restricted geographical ranges and occur as highly isolated populations [7].

Although, the effects of increasing salinity (secondary or anthropogenic salinisation) on freshwater ecosystems and saline lakes have been extensively reviewed, especially in Australia, producing a reduction in diversity [8-15], the contrary process, the reduction of salinity in naturally saline systems is less well documented [16]. In the Mediterranean area, the reduction of salinity in naturally saline systems has been an increasingly common phenomenon in recent decades due principally to changes in agricultural practices, such as the expansion of irrigated agriculture in the watersheds. Díaz et al. [16] emphasized the fragility of hypersaline lagoon ecosystems in the face to anthropogenic disturbances, such as increases in freshwater inflow and nutrient inputs.

The purpose of our study was to investigate the relationship between salinity and the biomass of primary producers, and the composition and structure of macroinvertebrate communities in a hypersaline stream, together with its influence on biodiversity at local and regional scales. Our hypothesis is that salinity is the principal factor affecting the structure of the community, and that an extended period of salinity reduction may increase the abundance and richness of species, but reduce the richness or abundance of the most halophilic species, resulting in a possible reduction in biodiversity at regional level.

## Methods

### Study area

**Figure 1:**
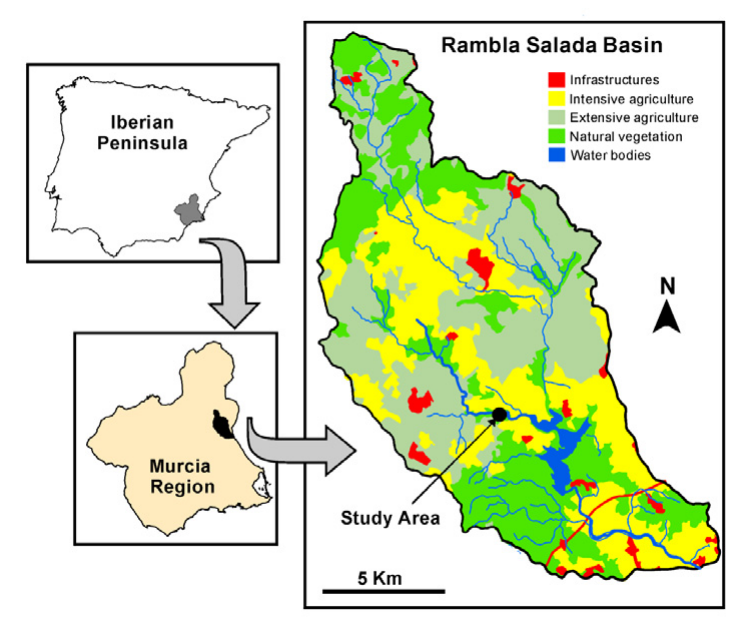
Geographic location of the Rambla Salada stream and land uses in the basin.

Rambla Salada is a Mediterranean hypersaline stream in the sedimentary Fortuna basin which belongs to the Segura River watershed (southeast of the Iberian Peninsula, Figure 1). The climate in the basin is semiarid with a mean annual temperature of 18°C and annual precipitation of around 250 mm. A long warm and dry summer season is interrupted by spring and autumn rains, the latter followed by a temperate winter. The watershed, of 4470 ha, is characterised by one main channel 11.6 km long and temporary tributaries that flow only during rainy periods. The main channel has permanent flow in its middle and lower sections, but flow is intermittent at the head. The natural cover on the watershed is open Mediterranean scrub, although much is dedicated to citrus and horticultural crops (intensive agriculture).

The high salinity in Rambla Salada is principally due to Miocene gypsiferous marls in the basin [17]. The ionic composition of the streamwater is dominated by Na^+ ^and Cl^- ^ions, followed by SO_4_^2- ^and Ca^+ ^[18].

**Figure 2:**
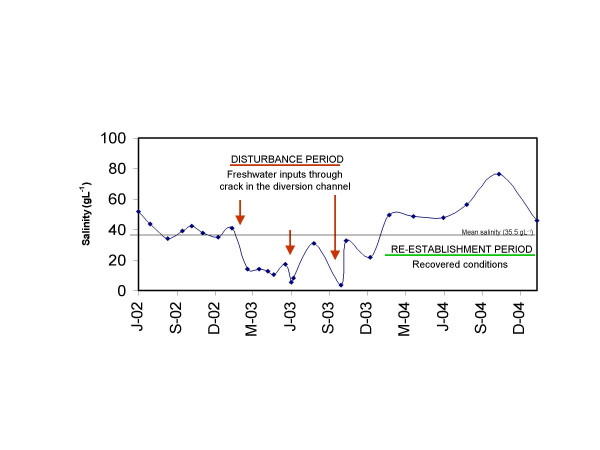
Salinity variation in Rambla Salada stream from June 2002 through January 2005, with disturbance and reestablishment periods indicated.

Since 1979 the Tajo-Segura river diversion has brought water for irrigation, and agriculture in the watershed has changed from the cultivation of extensive dry crops to intensively irrigated crops. There has been an increase in the input of freshwater, nutrients, pesticides and other pollutants derived from the crops into the stream. As consequence, since the 1980s, the salinity of the stream has fallen from close to 100 g L^-1 ^[19] to a mean of 35.5 g L^-1^. The diversion channel that crosses the stream suffers cracks at least once at year, which produce substantial discharges of freshwater into the stream. One such accident occurred in 2003 with the result that on 2 October the Rambla Salada stream received a discharge of 400 L.s^-1^, reaching its lowest recorded salinity level (3.5 g L^-1^) as the channel was emptied for repair (Figure 2).

The reference macroinvertebrate community dates from 1983, in the most hypersaline conditions before the important land use changes took place, but is only based on dispersed data of coleoptera and heteroptera species presence [20-24]. In addition, there is no control site upstream, because of the different conditions (lower salinity, temporary flow and human impact). Thus, taking into account the rapid community recovery in saline streams after flash flood disturbance [25], we have considered the re-established conditions of Rambla Salada stream one year after the crack disturbance as the state most similar to natural hypersaline conditions to investigate the effect of salinity changes on the composition and structure of biological communities.

### Sampling and processing of the samples

**Figure 3:**
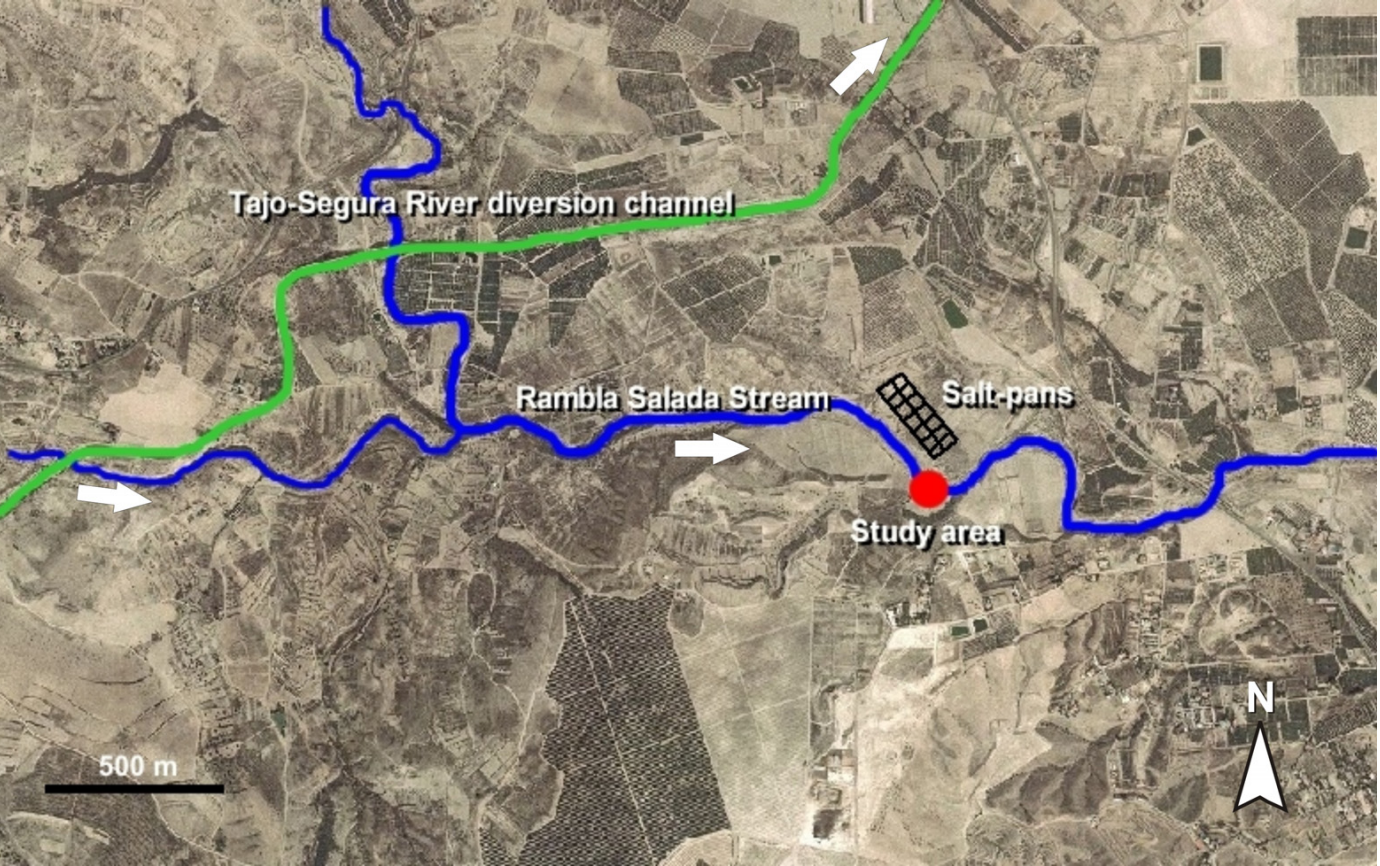
Aerial view of the Rambla Salada stream, showing the location of the study area, the abandoned salt-pans, and the Tajo-Segura river diversion channel.

**Figure 4:**
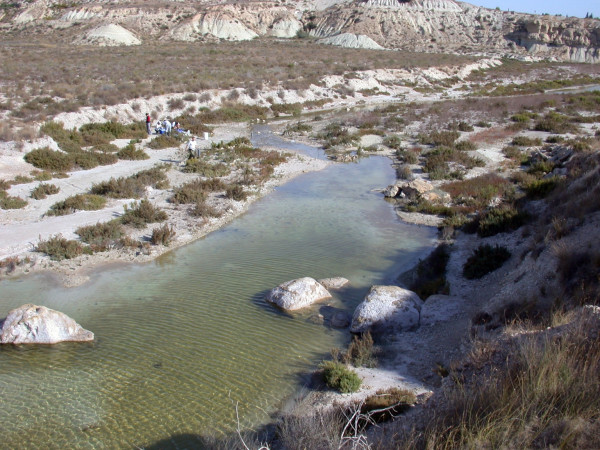
General aspect of the study area in Rambla Salada stream. October 2004.

**Figure 5:**
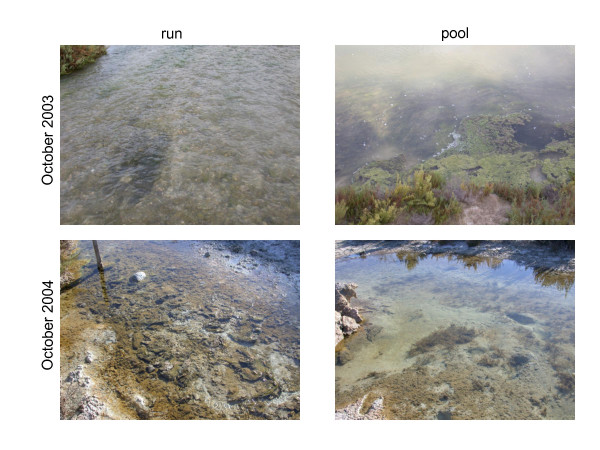
Run and pool habitats sampled in the Rambla Salada stream in October 2003 (3.5 g L^-1^) and October 2004 (76.4 g L^-1^).

To remove the seasonal variation component, sampling was performed on four dates: June and October 2003 during the dilution disturbance, and the same months in 2004 when the system had been reestablished. The four dates pointed to an in-site salinity gradient (3.5 – 76.4 g L^-1^). The study was made in the middle section of the stream, near an abandoned salt-pan exploitation (lat. 38°7'28"N, long. 1°6'46"W) (Figures 3 and 4). Pools and runs constitute the principal aquatic habitats in the reach (Figure 5). Shallow pools (≤ 60 cm) occupy more than 80% of the channel surface. They are characterised by large deposit of silts covered by a biofilm with diatoms and cyanobacteria as primary producers. Runs are characterised by gravel and sand as dominant substrates, are <10 cm deep and have a maximum water velocity of 0.37 m.s^-1^. This habitat was covered principally by the filamentous algae *Cladophora glomerata*.

On each date, water temperature and dissolved oxygen were measured in situ at 15 minute intervals during 24 hours using a multi-parametric recorder (WTW, MultiLine P4). Conductivity was measured in the morning with an ECmeter (TetraCon^R ^325) that automatically calculates salinity. Discharge was estimated from measurements of depth and current velocity along a cross-section in the run. Cover percentage of the different primary producers was visually estimated in each habitat.

In the run, three water samples were taken to measure suspended solids, chlorophyll *a *and dissolved nutrients (ammonium, nitrate, nitrite, and phosphate concentrations). The samples were kept cool until their arrival in the laboratory, where they were filtered onto preashed and preweighed GF/F glass-fiber filters. The filters were oven-dried at 60°C to constant dry weight and then ashed at 450°C for 4 h to estimate the input of suspended sediments (SS) and particulate organic matter (POM). Chlorophyll *a *concentration was determined by spectrophotometry, following extraction in 90% acetone. Ammonium was converted to ammonia by adding 10 M NaOH solution and measured with an ammonia electrode connected to a pH/mV meter. The rest of the dissolved inorganic nutrients were determined according to standard methods [26]: nitrate by the cadmium reduction method, nitrite by sulfanylic acid colorimetry, and phosphate by ascorbic acid colorimetry.

In both habitats, benthic macroinvertebrates were collected from the bottom using a 0.014 m^2 ^core. Substrate was removed by hand and a sieve (250 μm mesh size) was passed across the bottom and through the water column until no individuals were found. In lentic habitat, nektonic macroinvertebrates were also collected from the water column using a big core (0.1 m^2^). Five replicate cores per habitat were taken randomly on each sampling date, and material was preserved and stored in 75% ethanol until separation and identification in the laboratory.

Five benthic organic matter samples of sediments were collected by cores of 5.3 cm diameter and 24.5 cm length. Sediment samples were dried at 60°C to constant dry weight and then ashed at 450°C for 4 h to estimate the content of ash free dry weight (AFDW).

The biomass of the different primary producers in each habitat was estimated from five replicate samples using for macrophytes species the same core type as for sediments. For epipelic algae, we used a minicore of 2 cm diameter and 2 cm length. Samples were stored on ice and frozen until processing. Sixty milligrams of fresh macrophyte were taken from each sample to determine Chl *a *concentration by spectrophotometry, following extraction in acetone. The rest of the macrophyte was dried at 60°C to constant dry weight (DW). Epipelic samples were filtered onto Whatman A filters to determine Chl *a *concentration and AFDW.

### Data analyses

Macroinvertebrate data from each habitat were analysed both separately and combined to create a composite sample, representing one date.

The following structural macroinvertebrate community parameters were calculated: density, richness (number of total taxa, Diptera and Coleoptera species, and families). In addition, several diversity indices (Margalef's index, evenness, Shannon's and Simpson's diversity indices) were calculated using PRIMER (V.9). Comparisons were made using nonparametric Mann-Whitney U paired tests to compare differences between disturbed and re-established conditions in a given month. Spearman rank correlation was used to analyse the relationships of each community parameter with the salinity. Statistical analyses were performed using STATISTICA (V.6). An analysis of similarity (UPGMA, Bray-Curtis distance metric) was used to compare differences in macroinvertebrate communities between habitats and under the different salinity conditions. Finally, we used canonical correspondence analysis (CCA) with a down weight of the rare species to extract patterns of variation in macroinvertebrate communities in relation to environmental variables. Multivariate analyses were performed using MVSP (V.3.1) after log (x+1) data transformation.

## Results

### Physical and chemical environment

Water temperatures were slighter higher during 2004 than 2003 for the same months. The highest discharge value was reached in October 2003 due to large freshwater inputs from the Tajo-Segura channel, while the minimum discharge was reached in October 2004 after a long drought period. Salinity ranged from 3.5 to 76.4 g L^-1^, coinciding with the maximum and minimum discharge values (Table 1). Water was well oxygenated in the morning on all dates due to high benthic primary production, although night values of dissolved oxygen were low due to high ecosystem respiration (unpublished data). Mean daily percentage of oxygen saturation exceed 100%.

**Table 1:** Mean of the water quality parameters on each sampling date. Data for the natural hypersaline reference state were obtained from Vidal-Abarca (1985).

	2003 disturbed conditions		2004 re-established conditions		1983 Reference conditions
	
Water quality variables	J3	O3	J4	O4	July
Maximum daily temperature (° C)	31.4	26.2	33.7	29.3	35
Minimum daily temperature (° C)	20.1	19.2	20.3	12.3	
Mean daily temperature (° C)	25.1	21.9	25.5	19.4	
Discharge (L s^-1^)	31.48	262.16	33.47	13.23	
Conductividad (mS cm^-1^)	11.81	6.4	69.3	99	170
Salinity (g L^-1^)	6.8	3.5	47.5	76.4	100
Maximum dissolved oxygen (mg L^-1^)	16.45	23.1	16.78	18.17	
Minimum dissolved oxygen (mg L^-1^)	2.22	5.89	2.3	4.91	
Mean oxygen saturation (%)	116.43	111.7	143.69	152.98	
Suspended solids (mg L^-1^)	1.63	8.15	3.64	5.5	
Chlorophyll *a *(mg L^-1^)	4.22	1.52	25.12	0.83	
NO_3_-N (mg L^-1^)	0.22	1.98	1.47	0	1.13
NO_2_-N (mg L^-1^)	0.018	0.011	0.23	2.34	0.1
NH_4_-N (mg L^-1^)	0.25	0.15	0.89	2.69	0.63
DIN (mg L^-1^)	0.49	2.14	2.59	5.03	1.86
PO_4_-P (μg L^-1^)	8.68	10.78	0	1.61	0.5

The availability of total dissolved inorganic nitrogen (DIN) was higher than phosphate availability throughout the study period. The dominant form of nitrogen varied with salinity. High amounts of reduced forms (NH_4_^+^and NO_2_^-^) were registered in October 2004, when the highest salinity value was reached; while the oxidized form (NO_3_^-^) predominated at all other times. Phosphate concentrations were higher on the disturbed dates than on the reestablished dates. The nutrient levels measured in July 1983 [19], considered as the natural hypersaline reference conditions, were lower than those observed during the studied period, although very close to that registered in June 2004 (Table 1). Maximum chlorophyll *a *concentration in water was reached in June 2004, and no clear relationship was noted between this parameter and nutrient availability and salinity.

**Figure 6:**
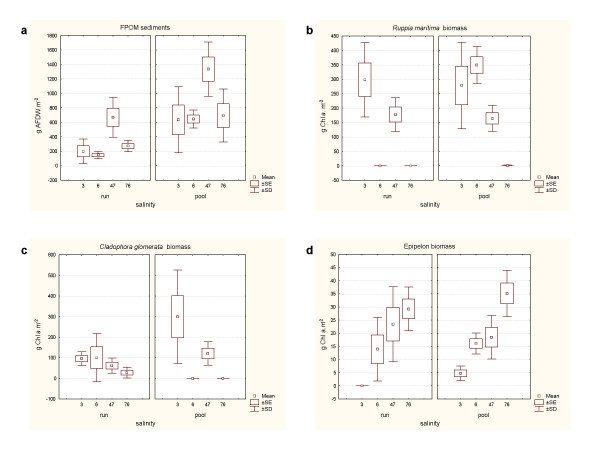
Variation in run and pool habitats of the content of fine particulate organic matter of sediments (a) and biomass of *Ruppia maritima *(b), *Cladophora glomerata *(c) and epipelic algae (d) in relation to the salinity gradient.

Sediments were rich in fine particulate organic matter (FPOM) on all the dates, especially in the pool habitat. The highest values were reached in June 2004, both in pool and run habitats (Figure 6a). There were significant differences between both June samplings but not between October samplings (Table 2). No significant correlation was found between FPOM and salinity (Table 3).

**Table 2:** P-levels of significance of the Mann-Whitney U paired test for comparisons between disturbed and re-established conditions in a given month.

	combined habitats	
	
	J3–J4	O3–O4
**Organic matter sediments**	**	n.s.

**Biomass primary producers**		
*Ruppia maritima*	n.s.	**
*Cladophora glomerata*	n.s.	**
Epipelic algae	n.s.	**

**Macroinvertebrate community parameters**		
number of taxa	**	*
number of families	n.s.	n.s.
number of diptera species	**	n.s.
number of coleoptera species	n.s.	n.s.
log (abundance +1)	n.s.	n.s.
Margalef's index	n.s.	*
evenness	n.s.	n.s.
Shannon's diversity index	n.s.	*
Simpson's diversity index	n.s.	n.s.

*P < 0.05, **P < 0.01

**Table 3:** Spearman correlation coefficients relating salinity and primary producers biomass and organic content of sediments, (n = 20).

Variable & salinity	R	p-level
Organic matter sediments	0.264	0.261
*Biomass of Ruppia maritima*	**-0.760**	**0.000**
*Biomass of Cladophora glomerata*	**-0.605**	**0.004**
Biomass of epipelic algae	**0.869**	**0.000**

### Primary producers

During the study period, primary producers in the stream were represented by an extended biofilm containing diatoms and cyanobacteria that covered the sediments, the filamentous algae *Cladophora glomerata *and *Enteromorpha intestinalis*, and the widgeon-grass *Ruppia maritima*.*Enteromorpha intestinalis *covered less than 5% of the channel and was not sampled for the estimation of biomass. The three variables studied (coverage, biomass (gAFDW.m^-2 ^and gChl *a *m^-2^) showed high correlation coefficients for each primary producer.

In general, the biomass of macrophytes was higher in the pool than in the run habitat (Figure 6b–c). The biomass of *R. maritima *was significantly different between dates (Table 2), Chl *a *concentration being higher in 2003 (disturbed conditions) than in 2004 (reestablished conditions). *Cladophora glomerata *showed significant differences in biomass between the October months, being much higher in the more dilute conditions. However, the biomass of epipelic algae was significantly higher in October 2004 than in October 2003, but no significant differences were found between the June months (Figure 6d and Table 2). *Cladophora glomerata *and *R. maritima *showed a significant decrease in Clh *a *content with increasing salinity, while the biomass of epipelic algae increased (Table 3 and Figure 6b–d).

### Macroinvertebrates

The macroinvertebrate community was characterized by low species diversity. A total of 25 taxa representing 16 families were recorded in the Rambla Salada stream during the study period (Table 4). Diptera and Coleoptera were the richest insect orders, with 10 and 9 species, respectively. Total taxon richness ranged from 11 to 15 in the run, and from 9 to 17 in the pool.

**Table 4:** Macroinvertebrate taxa occurring in the study area, with indication of their presence in the different habitats, and in the hypersaline reference community (REF 1983); mean density of macroinvertebrate taxa during the study period, and P-levels of significance of the Mann-Whitney U paired test for comparisons between disturbed and re-established conditions for a given month.

				Habitat		REF 1983	Mean density (ind m^-2^)				Mann-Whitney U test	
						
Order	Family/Subfamily	Genus/species	code	Run	Pool		J3	O3	J4	O4	J3–J4	O3–O4
Oligochaeta	Tubificidae		tubi	+					1035		**	n.s.
Gastropoda	Hydrobiidae	*Potamopyrgus antipodarun*	poan	+	+		1125	5124	657	972	n.s.	n.s.
Neotaenioglossa	Melanopsidae	*Melanopsis praemorsa*	mela	+				7			n.s.	n.s.
Ephemeroptera	Baetidae	*Cloeon schoenemundi*	clsh	+	+		377	287	7	112	**	n.s.
Odonata	Aeshnidae	*Anax sp*.	anax		+			14			n.s.	n.s.
Diptera	Chironomidae/Chironominae	*Chironomus salinarius*	chsa	+	+		38920	2978	3977	7640	n.s.	n.s.
	Chironomidae/Chironominae	*Chironomus sp*.	chir	+	+		210	7			n.s.	n.s.
	Chironomidae/Tanypodinae		tanp		+		245	14			n.s.	n.s.
	Chironomidae/Orthocladiinae	*Halocladius varians*	hava	+	+		2691	538	4592	329	n.s.	n.s.
	Ceratopogonidae		cera	+	+		105	7			**	n.s.
	Culicidae		culi		+			7		7	n.s.	n.s.
	Ephydridae	*Ephydra flavipes*	epfl	+	+	+	203	105	49	3940	n.s.	**
	Dolichopodide		doli		+					7	n.s.	n.s.
	Stratiomyidae	*Stratiomys longicornis*	stlo	+	+	+	7	7	21	147	n.s.	n.s.
	Simuliidae		simu	+			147	336			n.s.	*
Heteroptera	Corixidae	*Sigara selecta*	sise		+	+	3942	1014	804	126	n.s.	**
Coleoptera	Dytiscidae	*Nebrioporus ceresyi*	nece	+	+	+	391	217	21	7	**	n.s.
		*Nebrioporus baeticus*	neba	+	+	+	259	391	112		n.s.	**
	Hydrophilidae	*Berosus hispanicus*	behi		+	+	28	189		75	n.s.	n.s.
		*Enochrus falcarius*	enfa	+	+	+	405	77	496	154	n.s.	n.s.
		*Enochrus bicolor*	enbi	+						7	n.s.	n.s.
	Hydraenidae	*Ochthebius cuprescens*	occu	+	+	+	147	84	161	28	n.s.	n.s.
		*Ochthebius delgadoi*	ocde	+			7	14	7	14	n.s.	n.s.
		*Ochthebius tudmirensis*	octu	+			7		7		n.s.	n.s.
		*Ochthebius corrugatus*	occo	+	+		7			496	n.s.	**

*P < 0.05, **P < 0.01

### Individual taxon response

Eight of the species collected were known to be essential components of the natural hypersaline reference community of Rambla Salada from 1983 and were abundant during the studied period, both in disturbed and re-established conditions: *Ephydra flavipes *and *Stratyomis longicornis*, among the dipterans; *Nebrioporus ceresyi*, *N. baeticus*, *Berosus hispanicus*, *Enochrus falcarius *and *Octhebius cuprescens*, among the beetles, and the corixid *Sigara selecta*. However, some halophilic beetles species referenced from 1983, such as *Ochthebius glaber*, were restricted to a small hypersaline source outside of the study area, or were absent, such us *O. notabilis *and *Enochrus politus*.

Other species, such as the chiromomids *Chironomus salinarius *and *Halocladius varians*, the gastropod *Potamopyrgus antipodarum *and the mayfly *Cloeon schoenemundi*, were also abundant along the salinity gradient studied, although there is no information about their presence in the reference conditions. However, the freshwater species *Melanopsis praemorsa *and *Anax sp*. only appeared in low numbers in the Rambla Salada stream during the lowest salinity conditions, while *Dolichopodidae *and *Enochrus bicolor *appeared in the highest salinity level. Other dipterans, such as Ceratopogonidae, Simulidae, Tanypodinae and *Chironomus *sp., were found only in disturbed conditions.

*Nebrioporus baeticus *and *S. selecta *showed significant differences in abundance between the October months, being higher in the more dilute conditions (Table 4). However, the abundance of *E. flavipes *was significantly much higher in October 2004 than in October 2003. *Nebrioporus ceresyi *and *C. schoenemundi *were significantly more abundant in June 2003 than in June 2004.

The abundance of *Anax *sp., *Simulidae*, *S. selecta*, *N. ceresyi*, *N. baeticus*, and *B. hispanicus *showed significant negative correlations with salinity, while *E. flavipes *and *O. corrugatus *showed significant positive correlations (Table 5). The rest of species showed no significant abundance response to the salinity changes.

**Table 5:** Spearman correlation coefficients relating salinity and abundance of macroinvertebrate taxa, (n = 20).

Taxon abundance & salinity	R	p-level
Tubificidae	0.222	0.347
*Potamopyrgus antipodarun*	-0.198	0.404
*Melanopsis praemorsa*	-0.308	0.187
*Cloeon schoenemundi*	-0.418	0.067
*Anax sp*.	**-0.447**	**0.048**
*Chironomus salinarius*	0.074	0.757
*Chironomus sp*.	-0.292	0.214
*Tanypodinae*	-0.327	0.159
*Halocladius varians*	-0.062	0.795
Ceratopogonidae	-0.369	0.109
Culicidae	0	1
*Ephydra flavipes*	**0.576**	**0.008**
Dolichopodide	0.308	0.187
*Stratiomys longicornis*	**0.519**	**0.019**
Simuliidae	**-0.656**	**0.002**
*Sigara selecta*	**-0.644**	**0.002**
*Nebrioporus ceresyi*	**-0.473**	**0.035**
*Nebrioporus baeticus*	**-0.772**	**0.000**
*Berosus hispanicus*	**-0.531**	**0.016**
*Enochrus falcarius*	0.179	0.451
*Enochrus bicolor*	0.308	0.187
*Ochthebius cuprescens*	-0.150	0.527
*Ochthebius delgadoi*	0	1
*Ochthebius tudmirensis*	0	1
*Ochthebius corrugatus*	**0.708**	**0.000**

### Community response

**Figure 7:**
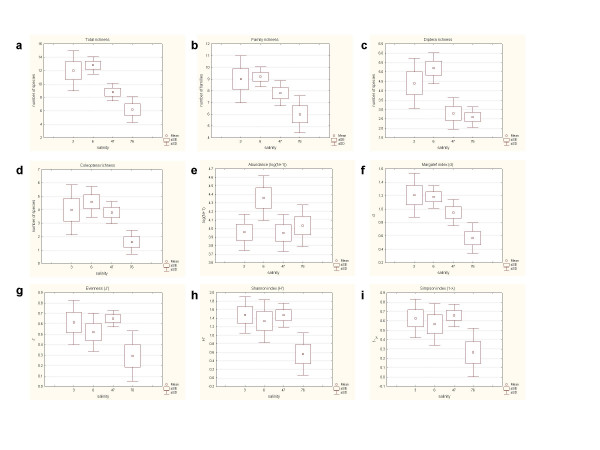
Variation of different composition and structural macroinvertebrate community parameters in relation to the salinity gradient.

The number of taxa, Margalef's index and Shannon's index differed significantly between the extreme conditions of the salinity gradient (Figure 7 and Table 2). Values of these indices were significantly higher in disturbed than in reestablished conditions. Significant changes in the number of Diptera and total species were also observed between both summer months. Macroinvertebrate communities showed a significant increase in richness, Margalef's index and Shannon's diversity index with decreasing salinity (Table 6). However, the rest of the community parameters, such as the abundance of individuals, evenness and Simpson's index, showed no significant response to the salinity changes.

**Table 6:** Spearman correlation coefficients relating salinity and macroinvertebrate community parameters, (n = 20).

Community parameter & salinity	R	p-level
number of taxa	**-0.762**	**0.000**
number of families	**-0.649**	**0.002**
number of diptera species	**-0.636**	**0.002**
number of coleoptera species	**-0.569**	**0.009**
log (abundance +1)	-0.039	0.871
Margalef's index	**-0.729**	**0.000**
evenness	-0.333	0.150
Shannon's diversity index	**-0.527**	**0.017**
Simpson's diversity index	-0.442	0.051

**Figure 8:**
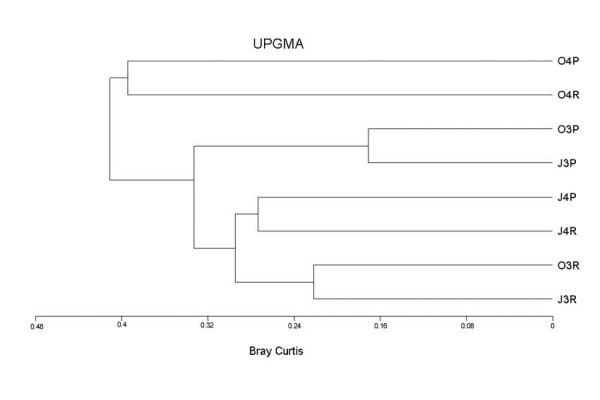
Bray-Curtis dendrogram based on macroinvertebrate assemblages. Samples are labelled with three digits: month, year, and habitat (R: run, P: pool).

The analysis of similarity, which compared communities by habitat, showed the greatest differences in macroinvertebrate assemblages between October 2004 and the rest of dates (41% of dissimilarity distance, Figure 8). In October 2004, the distance between both habitats was 39.5 %. On the disturbed dates, the differences between habitats were greater than between salinity levels (3.5 – 6.8 g L^-1^). Samples from June 2004, showed an intermediate position, reflecting their position in the salinity gradient, with 27.4% of distance between habitats.

**Figure 9:**
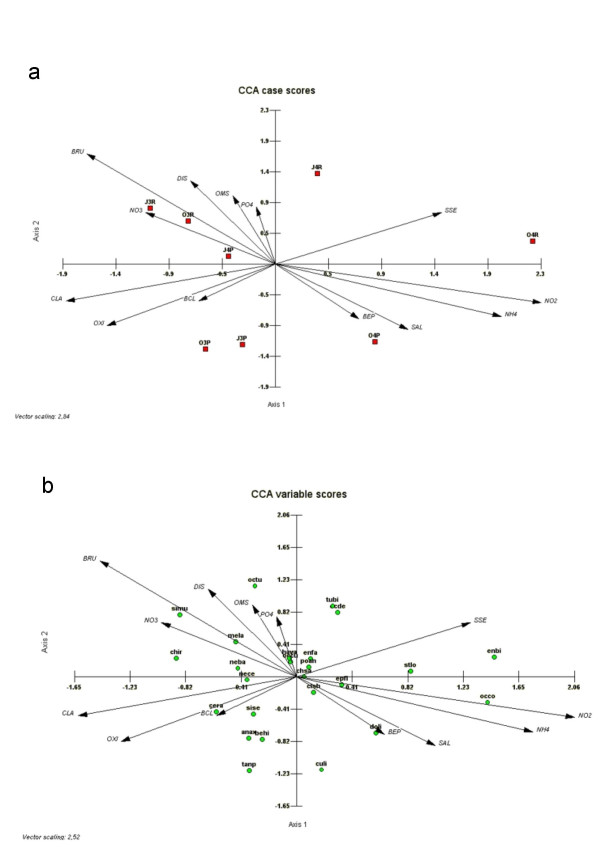
Canonical correspondence analysis plots representing samples and environmental variables (a), and macroinvertebrate species and environmental variables (b) on first and second axis. Samples are labelled with three digits: month, year, and habitat (R run, P pool). Species are labelled with codes (see Table 4). Codes of environmental variables: BCL, biomass of *Cladophora*; BRU, biomass of *Ruppia*; BEP, biomass of epipelon; SSE, suspended sediments; OMS, organic matter of sediments; DIS, discharge; OXI, dissolved oxygen; and CLA, concentration of chlorophyll *a *in water.

Canonical axes from the CCA analysis pointed to a high percentage of variability in the samples, with 28 % on the first axis and 27 % on the second. The first axis was related to the salinity gradient and differentiated samples from October 2004, when the highest salt water concentration was reached, from the rest of samples (Figure 9a). Besides salinity, this axis was positively related to ammonium, nitrite and suspended sediments, and negatively associated to dissolved oxygen, water Chl *a *concentration and *R. maritima *biomass. The second axis differentiated between samples from the run (positive scores) and pool (negative scores) habitats. The second axis was associated positively with discharge and biomass of *R. maritima*.

Species with a wide range of salinity tolerance and present in both habitats (pools and runs) appeared near the centre of the variable scores plot (Figure 9b), while the most halophilic species appeared at the positive extreme of the first axis, for example *E. bicolor *in the runs and *O. corrugatus *in both habitats. Species in the top-left hand corner of the diagram, *O. tudmirensis*, *M. praemorsa *and simulids, are restricted to the run habitat, while *Anax *sp.and Tanypodinae, which preferred pools, lie in the bottom-left hand corner of the plot.

## Discussion

Salinity changes ecosystem functioning by altering abiotic and biotic processes [13]. Changes in salinity affect aquatic biota directly or indirectly. Salinity can influence, for example, pH, dissolved oxygen and the nutrient balance of plants. It may be that biota respond as much to these indirect effects as to the direct ionic effects of salt concentration [27]. In our study, salinity had a strong effect on the biomass of primary producers in Rambla Salada stream, the biomass of *C. glomerata *and *R. maritima *increasing as salinity levels fell, while the biomass of epipelic algae decreased. Dilution changed the ecosystem's state from epipelic algae dominated to filamentous algae dominated. This result was expected because macrophytes are often absent or present in insignificant amounts at high salinity values [28]. In saline lakes, Wolheim and Lovvorn [29] found that aerial cover and both the species and structural diversity of macrophytes often decline as salinity increases. Although both the macrophyte species studied are halotolerant and are frequently found in mesosaline waters, they were adversely affected when salinity exceeded 75 g L^-1^. *Ruppia maritima *has specialized features, such as epidermal leaf cells modified to absorb both cations and anions for osmoregulation [30,31], which enable it to survive in a variety of saline conditions and at high temperature beyond those tolerated by other submerged angiosperms [32]. Widgeon-grass on the other hand, turns white and dies when exposed to high temperatures and widely varying salinity levels, because of the additional energy required for increased osmoregulation [31]. The same plant adapts poorly to turbid waters or anaerobic sediments [32], conditions prevalent in Rambla Salada stream in October 2004, when its biomass decreased significantly. Changes in macrophytic production with decreasing salinity might result from a combination of lower osmoregulation costs and greater nutrient availability. The high discharge and large input of freshwater would have mixed the sediments and associated nutrients within the water column and increased phosphorous availability. In the disturbed diluted conditions, high nitrate and phosphate concentrations favoured *C. glomerata *and *R. maritima *growth. Mason [33] found that the high productivity of *C. glomerata *was directly associated with high nitrate and high biochemical oxygen demand in farm ponds. However, ammonia and nitrite dominated in the hypersaline conditions of Rambla Salada stream and could have been the principal N source for epipelic algae. Although a diversity study of the phototrophic microbial community was not undertaken, in hypersaline conditions, high species diversity is expected, as Major et al. [34] found in Salt Plains of Oklahoma (USA).

On the other hand, because the form of primary production can change with salinity, involving shifts in the relative abundance of open-water and macrophyte/epiphyte habitats, indirect changes in macroinvertebrate communities are also to be expected [35]. These new habitats can support additional invertebrate community components [36]. *Cladophora glomerata *provide attachment sites for epiphytes, which can serve as food source for invertebrate grazers [37], besides supplying a refuge from predation [38].

**Figure 10:**
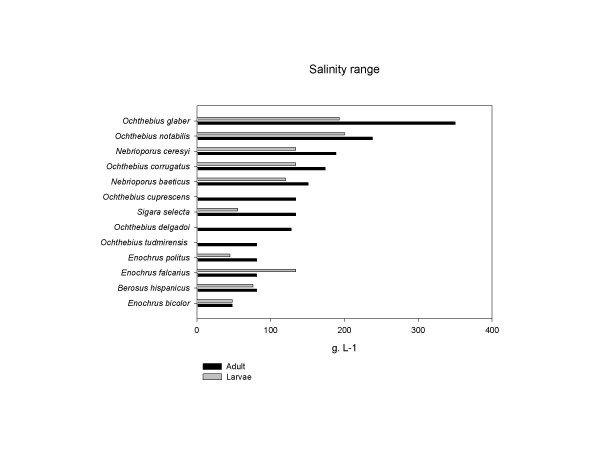
Salinity tolerance for Coleoptera and Heteroptera species recorded at past and recent dates in the Rambla Salada stream, obtained from distributional data for the aquatic systems of the Segura River basin.

The direct effects of salinity changes on macroinvertebrate community depend to a great extend on the salinity tolerance range of the species concerned [39]. Due to the absence of studies of osmotic regulation and salinity tolerance of the macroinvertebrate species occurring in Rambla Salada stream, and because there is no salt sensitivity database of Paleartic species as there is for Australian biota [27], salinity tolerance must be inferred from the maximum salinity at which species were collected. For Coleoptera and Heteroptera species, the upper salinity levels were obtained from a database of field collections carried out since 1980's in the Segura River basin, which comprises aquatic systems across a wide salinity gradient (Figure 10). For the rest of the taxa, we obtained information of the upper salinity levels of the family, genera or species (if possible) from published literature.

Dipterans and coleopterans showed the greatest diversity in the saline stream studied like as in saline lakes [40]. Among the dipterans showing the highest salinity tolerance, Ephydridae and Dolichopodidae families are known to be present in saline lakes up to 118 g L^-1 ^of salt concentration [41]. Chironomids have been collected at salinities greater than seawater [40]: for example, *C. salinarius *and *H. varians *that are found frequently in brackish waters in coastal lagoons and salt marshes [42,43]. Among Coleoptera, Hydraenids, Dytiscids and Hydrophilids contain species that lives in broad ranges of salinity. The genus *Ochthebius *has a great number of halophilic species, *O. glaber *exhibiting the highest salinity tolerance, being present throughout the entire salinity range and able to inhabit saturated brine (Figure 10). This species is followed in decreasing order of salinity tolerance by *O. notabilis, O. corrugatus*, *O. cuprescens*, *O. delgadoi *and *O. tudmirensis*. Among ditiscids, *N. ceresyi *and *N. baeticus *larvae have been recorded at salinities up to 100 and the adults to up to 150 g L^-1^. Among the hydrophilids, *E. falcarius *and *B. hispanicus *are the two more abundant species in mesosaline waters (~20 – ~50 g L^-1^), although have been found living at 81 g L^-1^. *Enochrus bicolor*, a halobiont species that can be found sporadically in Rambla Salada stream, is confined to saltmarsh/brackish water in the UK in a salinity range of 4.7–62.6 g L^-1 ^[44]. The corixid *S. selecta*, typical species of both brackish and inland saline waters [45], was found in salinities ranging from 3.5 to 134 g L^-1^, completing its life cycle up to 55 g L^-1^.

Mayflies are generally restricted to freshwaters, but some Baetidae species are common in moderately saline lakes [40]. *Cloeon schoenemundi *has been found both in fresh and hyposaline streams of semiarid areas of the Segura River watershed [46] and in Rambla Salada stream it occurred up to 75 g L^-1^, the maximum registered for any mayfly.

The New Zealand mud snail, *P. antipodarum*, is highly tolerant to salinity and resistant to desiccation [47]. These characteristics, together with its asexual reproduction and high dispersal capacity, have allowed the snail to invade streams, rivers and estuaries across Europe and west North America.

In our study, decreasing salinity resulted in an increased richness and diversity of macroinvertebrate species. The dilution of Rambla Salada stream allowed the colonization of freshwater species or low salinity tolerant species, such as *M. praemorsa*, or some *Anax *sp. Simulidae, Ceratopogonidae and Tanypodinae species. The most euryhaline species were the least affected by the dilution process, and were present both in disturbed and reestablished conditions. Three responses to salinity dilution were observed: i) an increase in the abundance of *S. selecta*, *N. ceresyi*, *N. baeticus*, and *B. hispanicus*, ii) a decrease in the abundance of *E. flavipes and S. longicornis *and iii) no significant difference in the number of individuals of *P. antipodarum*, *C. salinarius*, *H. varians*, *C. schoenemundi*, *O. cuprescens *and *O. delgadoi*. Most of these euryhaline species were the principal components of the macroinvertebrate community of Rambla Salada stream in past and recent years, when salinity varied from 100 to 3.5 g L^-1^.

The conceptual model developed by Herbst [48] to explain the abundance of *Ephydra hians *along a salinity gradient is useful for understanding species response to salinity dilution. This model proposes that abundance is maximal at salinity levels midway between the physiological limitations of high-salinity stress and the ecological limitations imposed by biotic interactions (e.g., predation and competition) at low salinity. On the other hand, the ability of many halophiles and halobionts to tolerate lower salinities in the laboratory than those to which they are exposed in the field suggests that biotic factors exclude them from habitats of lower salinity [40].

Several of the diversity indices changed along the salinity gradient. Total richness, the number of Diptera and Coleoptera species, the number of families, Margalef's index and Shannon's diversity index increased with decreasing salinity. However, the rest of community parameters, such as the abundance of individuals, evenness and Simpson's index, showed no significant response to changes in salinity. Higher densities and lower richness, diversity and evenness were found by Bunn and Davies [11] for benthic biota during river salinization when salinity exceeded 3 g L^-1^.

Our results were to be expected, since the macroinvertebrate diversity follows a trend parallel to that shown by other animals and plants: i) richness and diversity indices fall as conditions become more extreme [49], and ii) at lower salinities, most species are halotolerant while at higher salinities most of them are halophilic [50].

Although assemblages changed gradually along the salinity gradient, at a given level of salinity there was an abrupt and major change in the composition and structure of the macroinvertebrate community. The analysis of similarity confirms that significant changes toock place in the community structure once salinity exceeded 75 g L^-1^. Bellow this salinity level, difference in flow velocity, the organic content of sediments and the biomass of macrophytes between pool and run habitats had a greater effect on macroinvertebrate communities than salinity changes. Similarly, some authors have found that small changes in salinity are not well related to macroinvertebrate [51,52] and microbial [34] communities, because of the broad ranges of salinity and temperature tolerance of inhabitants of saline systems in arid and semi-arid environments. Streams in Mediterranean climate regions are influenced by a sequence of regular and often extreme flooding and drying periods and their inhabitants are adapted to extreme, often rapid, environmental change [53]. However, prolonged dilution or long-term effects of decreasing salinity by anthropogenic disturbances might result in substantial biological changes, and the most halophilic biota will be adversely affected as salinity falls below 75 g L^-1^. For example, *O. glaber*, an endemic halophilic species of high genetic diversity and great conservation interest [54,55] is known to have disappeared in recent decades from the study area, where it remains only in a small hypersaline source (~150 g L^-1^) in the Rambla Salada stream. *Ochthebius notabilis*, another hydraenid species proposed as vulnerable species in the red list of Spanish invertebrates [55], frequent in salt-pans and hypersaline pools habitats [56], has not been found in Rambla Salada stream since 1991 (unpublished data). Our results indicate that although diversity at local level increases with dilution, with the introduction of estenohaline species with low to moderate salinity tolerance and wide geographical ranges, there is a risk that the aquatic diversity at regional and global levels will be reduced as the most halophilic and vulnerable species in the Mediterranean area, which often have restricted geographical ranges and occur as highly isolated populations [7], are eliminated.

## Conclusion

This study contributes to our understanding of the response of hypersaline stream biota to dilution and adds information concerning the saline thresholds of primary producers and macroinvertebrates. Our results support the initial hypothesis that dilution causes an increase in richness and biotic diversity, but a reduction in the abundance or elimination of the most halophilic species. There is a critical level of ~75 g L^-1^when an abrupt and major change in the structure of the macroinvertebrate community occurs, but below which occasional changes in salinity do not greatly affect community structure. Nevertheless, more studies of saline streams are needed to refine and confirm this assertion. Changes in macroinvertebrate communities due to other factors, such as habitat type and biotic interactions will reduce the relevance of changes due to salinity. Chronic dilution may produce a loss of biodiversity at regional level.

## Competing interests

The author(s) declare that they have no competing interests.

## Authors' contributions

JV conceived and coordinated the study, participated in the design, field sampling, water, sediment and primary producer analyses, statistical analyses and drafted the manuscript. AM participated in the design of the study, field sampling, identification of macroinvertebrates, interpretation of data, and performed the multivariate analyses. JH performed the calculation of community parameters and statistical analyses. JH, CG, DS, PA and MR collected field data, participated in water, sediment and primary producer analyses and the separation and identification of macroinvertebrates. All authors revised critically the draft for important intellectual content and approved this final manuscript.
